# The Idiot in Fiction

**Published:** 1915-03

**Authors:** 


### The Idiot in Fiction.

Most primitive peoples have regarded the idiot as under special Divine favor, even as in some degree a means of communicating Divine commands to ordinary mortals. This idea has persisted, in more or less altered form to the present. In many works of fiction, the idiot is an important character, often playing almost a heroic role, occasionally he or she is represented as the incarnation of an evil force, still less often, as a practical source of danger. The typic ament of fiction is a wandering, uncontrollable, but altogether lovable and beneficent character. Popular conceptions are, to a large degree by the novelist and play-wright who, therefore, possess great power of good or evil. In their characteristic depiction of the ament, they are inculcating a highly dangerous popular conception. The unconfined insane person is now pretty generally realized to be a potential source of danger, however gentle he may seem. The person who has never reached full mentality is equally dangerous potentially. In fact, every few days, the press reports cases in which an apparently harmless idiot perpetrates some terrible crime. Sexual irregularities are notoriously common among persons who, having no full intelligence, are, therefore, unable to comprehend moral law to the full degree. Even if a particular idiot goes through life without committing any overt criminal act, the danger of procreating other defectives is a serious one. The only safe course, either for the welfare of society or of the ament himself or herself, is life long segregation in a properly conducted institution. It is the duty of the medical profession to promulgate this conception of the status of the mentally deficient.

It may be of interest to note that DeFoe, the author of Robinson Crusoe, advocated institutional care, at state expense for "naturals," i. e., idiots, late in the 17th century.

